# Reducing Medical Costs and Improving Quality via Self-Management Tools

**DOI:** 10.1371/journal.pmed.0040104

**Published:** 2007-04-17

**Authors:** Harold J DeMonaco, Eric von Hippel

## Abstract

The authors consider the advantages and limitations of self-management tools used for treating chronic illness.

In 2004, health-care providers in the United States consumed $US1.9 trillion, or about 16% of the gross domestic product. By 2010, the cost of health care is predicted to exceed 20% of the US gross domestic product [1]. The management of chronic diseases currently accounts for 70%–75% of health-care spending [2], and this proportion is likely to increase in the future. Unlike acute illness, chronic illness requires continuous and sometimes complex management over prolonged periods of time, with the goal of improving or stabilizing quality of life.

One of the proposals for reducing the costs and improving the quality of chronic care is for patients to become their own providers of medical care [2]. Empirical studies of a number of chronic diseases support the feasibility of such a proposal [3–5]. Such studies found that self-management produced higher quality outcomes at lower costs than conventional models of care.

Self-management is defined as: (1) engaging in activities that protect and promote health; (2) monitoring and managing symptoms and signs of illness; (3) managing the impacts of illness on function, emotions, and interpersonal relationships; and (4) adhering to treatment regimens [6]. In this Essay, we consider the advantages and limitations of self-management tools.

## “Toolkits” for Patient Self-Management

Researchers in the field of innovation management have coined the term “user innovation toolkits” to refer to integrated sets of specialized tools that enable end users of a product or service to develop or modify products for themselves [7]. For example, toolkits can enable non-specialists to develop custom products including complicated integrated electrical circuits for themselves, without any assistance from factory experts. The shifting of product and service development tasks to the consumer is a major trend in many fields today, because it cuts costs while at the same time increasing quality of, and user satisfaction with, the new product [8,9].

Shifting a problem-solving task to users via a toolkit lowers costs when users possess the information required to solve the problem in raw form—and when it is cheaper to transfer the tools needed to process that information to the user than it is to transfer the user's information to an expert.

This same economic logic can be applied to medical toolkits devised for a specific purpose—patient self-management of chronic medical conditions. Such toolkits offer patients the tools needed to enable them to process their personal “raw” disease-related data into information that helps them manage their own illness. The toolkit gives patients the information needed to: (1) appropriately recognize when to apply a prescribed or over-the-counter medical treatment; (2) apply the treatment; (3) receive feedback on the results of the treatment; and (4) make appropriate treatment adjustments without the involvement of a health-care specialist.

Several patient self-management tools are already in use [10], such as in the management of patients with type 1 diabetes. After diagnosis, physicians generally supply patients with a toolkit for diabetes self-management. In other words, patients are: (1) supplied with and taught to use a blood glucose monitor to determine their blood sugar level; (2) taught how to apply the treatment they need (via self-injection of insulin for elevated blood glucose levels or oral sugar for low blood glucose levels); (3) taught how to get feedback on the results of their treatment action (again via a blood glucose meter); and (4) taught how to correct and improve their disease self-management activities based upon this feedback. Studies have shown a low rate of adverse events related to diabetes self-management [11–13]. Clinician support for patient self-management appears to be an important predictor of the quality of clinical outcomes in patients with diabetes [14].

Compare and contrast this toolkit model of diabetes self-care with the conventional management of hypertension. Despite the fact that home blood pressure monitoring is readily available and the drugs used to treat hypertension have a wide margin of safety, most patients repeatedly visit their health-care provider for routine blood pressure monitoring and routine drug dosage adjustments. And yet home monitoring has been shown to improve blood pressure reductions and adherence to medication [15].

Why is there a disparity in the management of these two chronic diseases? In the case of type 1 diabetes, simple logic suggests that there is no other way to manage the disease. Patients must be involved in self-management due to the nature of the illness and the need for timely self-administered treatment. However, the pharmacologic treatment of hypertension allows the health-care delivery system to supply adequate treatment via a conventional relationship between health professional and patient.

## Self-Management Toolkits Are Technically Feasible

What factors are important for successful self-management of a chronic illness? The first is showing patients how to self-administer standard evaluation scales or perform simple assessments that are currently administered by health professionals.

The second factor is pre-prescription of medicine and dosage adjustments based on specific assessment results. Individual doctors currently apply such dosage adjustments in a routine manner for most of their patients given specified assessment results. For example, adjustment of medication for depression is often accomplished by means of a questionnaire administered by health-care professionals. This method is routinely used in randomized clinical trails [16,17]. Given specific reassessment results, many specialists have a standard “next step adjustment” in dosages or medications that they routinely apply for most of their patients. It may thus be feasible to give patients with depression a self-management toolkit involving: (1) a questionnaire that allows them to assess the severity of their illness and (2) a pre-prescribed plan for adjusting the dosage in response to results of the assessment.

In the case of most medications, optimal dosages vary from patient to patient and, for a specific patient, may also vary over time and according to variations in living conditions. The use of toolkits allows a process of trial and error as users modify their actions based on experience and feedback. As each user iterates, he or she can move towards the optimal dosage for their specific circumstances. Toolkits must be designed to support this range of user experimentation while at the same time indicating areas of danger.

**Figure 1:**
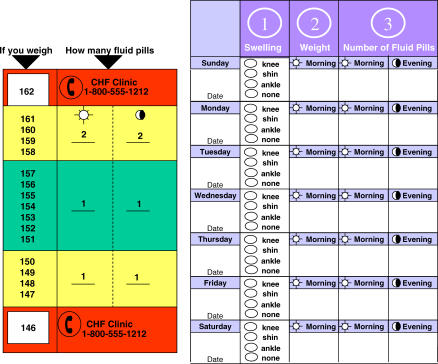
An Example of a Toolkit for All Literacy Level Patients with Congestive Heart Failure Adapted from [19].

There are data that suggest a well-designed toolkit may well reduce risks as compared to conventional care even in patients with low literacy [18,19]. Figure 1 is an excellent example of such a toolkit [19]. The toolkit allows patients to adjust their medication based on a systematic trial and error method. A critical element to this process is feedback. In this case, daily weights are used to adjust the dose of diuretic. This level of control would not be possible without the use of either the toolkit or the daily interaction with a health-care professional.

A research program to develop self-management tools, and to make them available as appropriate, must build on our existing knowledge of patient adherence to treatment. For example, patients are more likely to comply with a practitioner's instructions to take medicines as instructed when the patients can observe the impact of their behaviors [18–20]. Toolkits, with their feedback component, can be expected to improve adherence to a prescribed medication plan. Indeed self-management tools have been used to improve adherence to medication in patients with asthma [21].

## Why Have Toolkits Not Become Commonplace?

When chronic disease management becomes routine, as in the management of type 1 diabetes, it often becomes technically possible to shift management by professionals to self-care by patients equipped with appropriate tools and training. But even when such a shift becomes technically possible, there tends to be a lag in actually moving towards self-management. In good part this lag can be understood by considering what needs to happen at the level of health-care delivery systems for a change to occur.

Consider, for example, the use of anticoagulants on an outpatient basis. Several large clinical trails have shown that patient self-management (including home determination of the degree of anticoagulation and dosage adjustments) is as good as, if not better than, the care provided in specialized anticoagulation clinics [22,23]. In addition, self-management is significantly less costly to medical care insurance providers [24]. Indeed, both of these outcomes have been shown in large-scale practice in Germany, where anticoagulation self-management is now a routine practice [24,25].

Despite this evidence of better outcomes at lower costs via patient self-care, a recent survey of anticoagulation practitioners in the US indicated that only 1% of US patients on long-term anticoagulation regimens are involved in self-testing [26]. In fact, 60% of the specialist care providers who responded to the survey noted that patient self-care is specifically prohibited by the specialist's policies [26].

This situation is understandable when you consider that care provision systems designed around what was best practice in earlier days may well suffer a loss of business if they adopt newer practices. In addition, toolkits by their very nature tend to alter the conventional relationships between health professionals and patients, which could lead to professionals losing their status or even their jobs. The introduction of toolkits may therefore understandably be resisted unless they are introduced with sensitivity to these issues [27].

## Limitations and Recommendations

While self-management tools appear to have potential financial and clinical benefits, they also have limitations. By definition, toolkits can only be applied in situations where there is a clear and unambiguous feedback mechanism for patients. This feedback may take the form of a measurable symptom or a physiologic variable that is easy to monitor. To reduce the health risks associated with self-care, well-designed toolkits must allow the layperson to perform medical tasks at a level of sophistication nearly that of the expert. Although this might appear to be an impossible task, it has been achieved in many fields of medicine. For example, at least one recent review of evidence-based practices noted the value of patient self-management of anticoagulants as a risk reduction strategy [28].

We propose that the time has come for health systems to support appropriate and appropriately timed shifts from practitioner-based care to patient self-management. The use of toolkits in other fields has demonstrated an improvement in quality, lowering of costs, and more efficient completion of tasks. This trend holds even in very complex fields. We believe that this trend will also be evident in the management of patients with chronic illness.
